# Whose voice counts? Examining government‐donor negotiations in the design of Ethiopia’s large‐scale education reforms for equitable learning

**DOI:** 10.1111/dpr.12634

**Published:** 2022-07-19

**Authors:** Shelby Carvalho, Amare Asgedom, Pauline Rose

**Affiliations:** ^1^ Harvard University Cambridge USA; ^2^ Addis Ababa University Ethiopia; ^3^ REAL Centre, University of Cambridge UK

**Keywords:** education reform, Ethiopia, international aid, politics of aid, ownership

## Abstract

**Motivation:**

The Government of Ethiopia has a long‐standing commitment to improving the quality of education. In recent years, this has shifted to include a more explicit focus on equity in learning outcomes. In this paper, we examine the education reform design process in the context of Ethiopia’s political environment which is widely recognised as a strong developmental state.

**Purpose:**

The article examines how federal, regional, and international donor actors negotiate their interests in relation to Ethiopia’s national quality education reform programme, the General Education Quality Improvement Programme for Equity (GEQIP‐E).

**Methods and approach:**

We conducted 81 semi‐structured, key informant interviews with federal and regional government officials and international donors who were involved in the design of GEQIP‐E.

**Findings:**

We find that federal government was able to leverage considerable political influence over high‐level priorities and the framing of GEQIP‐E. Large donors leveraged financial influence to exclude some specific priorities, while smaller donors were able to draw on social influence and technical expertise to include priorities aligned with their interests. Regional governments—which are responsible for policy implementation—were largely excluded from the reform design process.

**Policy implications:**

Our analysis highlights the importance of recognizing and understanding different forms of influence in the dynamic process of negotiating reform between government and donors. It identifies that both government and donor voices counted in the process of negotiations, but in different ways and to varying degrees. Understanding how different actors draw on their relative political, financial, and social influence is vital for ensuring successful implementation and sustainability. Importantly, we identify that voices of local actors are left out.

## 1 INTRODUCTION

Improving educational quality with equity is a complex and often challenging task, particularly in large countries that encompass diversity. As a result, making such commitments can be logistically and politically complicated for policy‐makers. This may be particularly true in low‐income countries with limited domestic resources and an active international donor presence, resulting in potentially competing priorities. The article aims to examine the negotiation process of a policy commitment to ensuring quality with equity in Ethiopia’s education system. Specifically, we ask: How do federal, regional, and international donor actors negotiate their interests in relation to Ethiopia’s national quality education reform programme? Situating our analysis in the context of Ethiopia’s large‐scale education reform, we draw on key informant interviews with federal and regional government officials and donors in Ethiopia to examine “whose voice counts” in negotiations over the design of Ethiopia’s General Education Quality Improvement Programme for Equity (GEQIP‐E) (World Bank, 2017).

Following decades of civil war, severe drought, and famine, Ethiopia has achieved impressive economic growth and poverty reduction over the past 20 years (Clapham, 2018; Furtado & Smith, 2008; Lie & Mesfin, 2018). Between 2004 and 2014, real gross domestic product (GDP) grew by an average of more than 10% per year (Seid et al., 2016). Ethiopia also made significant strides towards the United Nations’ Millennium Development Goal (MDG) of achieving universal primary education (Lie & Mesfin, 2018). According to official statistics, net primary enrolment in the country increased from 21% in 1996 to 93% by 2014 (National Planning Commission & UN Ethiopia, 2015).

Education has remained a strong priority for the government following the adoption of the Sustainable Development Goals (SDGs, 2018). It features prominently in the government’s overarching development strategy to transition to a knowledge‐based economy and achieve middle‐income status by 2025 (National Planning Commission, 2016). This commitment to education is reflected by sizeable investments from the central government: in 2015, Ethiopia dedicated more than 27% of total public spending to the education sector (United Nations Department for Economic and Social Affairs, 2019), compared to less than 17% on average in the region.

Despite ambitious goals, substantial economic progress, and improvements in education access over the past 20 years, primary school completion and learning outcomes have fallen short, particularly for the most disadvantaged. According to 2016 Demographic and Health Survey (DHS) data, only around a quarter of the poorest 20% complete primary school. By extension, very few of these children enter secondary school, and just 4% complete this cycle (World Inequality Database on Education database, 2018). Student learning outcomes also remain low (Iyer et al., 2020).

Since 2008, Ethiopia’s education reform agenda has been guided by the government’s General Education Quality Improvement Programme (GEQIP). This reform effort is supported by donors, notably the World Bank, the United Kingdom’s Foreign, Commonwealth and Development Office (FCDO, formerly the Department for International Development, DFID), the governments of Finland and Norway, and UNICEF. While the main goal of the GEQIP reforms has always been to improve education quality, the approach to addressing quality has evolved, moving from a focus on inputs (GEQIP I, 2008‐13), to processes (GEQIP II, 2013‐17) and, most recently, to a specific focus on learning outcomes along with explicit attention to equity (GEQIP‐E, 2018‐23, with “E” referring to equity). This shift coincides with the SDG’s emphasis on ensuring “inclusive and equitable quality education.”

As a programme designed through close collaboration between the Government of Ethiopia and donors, GEQIP presents an important case study of collaboration and negotiation processes between education stakeholders working towards the design of a complex reform agenda for quality education with equity. We add to the small but growing literature on government–donor negotiations in education reform processes, providing insights into how actors leverage different sources of influence—including political, financial, and social—over the policy‐making process. We find that the federal government was able to leverage considerable political influence over high‐level priorities and the framing of GEQIP‐E in particular, while donors leveraged financial or social influence to shape specific policies and activities that were included or excluded from GEQIP‐E’s design.

This research was conducted before the COVID‐19 pandemic and prior to the start of the conflict which began in late 2020 in Ethiopia. GEQIP‐E remains the main education reform effort for improving the quality of primary schooling in the country and continues to move forward in regions not involved in the conflict. While the specific goals and implementation of GEQIP‐E have slowed due to ongoing crisis and uncertainty, the findings of this research remain relevant in Ethiopia, particularly those highlighting regional exclusion from policy negotiations. Findings related to policy negotiation, influence, and ownership dynamics between government and donors are also relevant for other large aid‐recipient countries around the world.

## 2 CONCEPTUAL FRAMEWORK

To examine how education policy influence is leveraged and varies across actors in Ethiopia’s large‐scale quality education reform, it is helpful to understand the broader political and institutional context. In the political settlement literature, Ethiopia has been described as a “*dominant developmental*” state, with cohesive intra‐elite relations within the ruling coalition and strong government control over resources and decision‐making (Clapham, 2018; Hickey & Hossain, 2019; Yorke et al., 2021). Despite being a federal system, the central government maintains control over policy priorities with limited pressure to respond to outside constituencies. Most existing approaches to studying the political economy of education reform are difficult to apply to the Ethiopian context. In particular, the emphasis in existing frameworks on teachers’ unions, collective action, and the private sector in driving education investments and priorities are not directly applicable. For example, due in large part to the nature of Ethiopia’s political and economic environment, it is less likely that significant pressure would arise from civil society or from the relatively small teachers’ union, which are noted as important drivers of reform in other contexts (see for example, Bruns et al., 2019; Kosack, 2012).

Our work contributes to the growing literature on ownership and international aid (Booth, 2012; Menashy, 2019; Swedlund, 2017; Swedlund & Lierl, 2019; Whitfield & Fraser, 2008a). While greater ownership by recipient countries over international aid has become a fundamental principle for improving aid effectiveness in multiple international strategies and declarations,^1^ the degree of recipient‐country ownership varies in practice, due to diverse factors including state capacity and regime type. Whitfield and Fraser (2008a) argue that there are two competing, potentially contradictory, forms of recipient‐government ownership in the aid encounter: ownership as a commitment to accept donor policies (“an obligation to accept responsibility for implementing them”), and ownership as control over the process and outcome of choosing policies (“the degree of control recipient governments are able to secure over implemented policy outcomes”, 2008a, pp. 3–4). While these concepts of ownership as commitment and control are useful, they imply a zero‐sum game, in which one party can claim ownership and others cannot. Swedlund and Lierl (2019) argue that aid policy should instead be thought of as the result of sustained negotiations between governments and donors over time, in which compromises are made to maximize utility for both parties, as opposed to being thought of in terms of more absolutist ideas of ownership and control.

The degree to which aid‐recipient governments exercise ownership over the policy process is partially dependent on the leverage that a negotiator is able to derive from their political, financial, social, and institutional influence within which donors and recipients define their preferences and select their strategies (Whitfield & Fraser, 2008b, p. 39). The ability of governments to exercise ownership can also depend on the nature of aid. At one end of the spectrum, traditional, project‐based aid typically allocates money for specific purposes with limited government influence in the design, while at the other end, aid is linked to wider government policy reforms or actions, such as through pooled funds. The latter, which is more aligned with the pooled‐funding approach of GEQIP‐E, can necessitate compromise between, or prioritization of some interest groups over others, both among donors themselves, and between government and donors. In reality, donors and governments are often engaged in what Swedlund (2017) terms “the development dance,” where donors and governments each face difficulties in making credible commitments to each other, because each has their own set of priorities, roadblocks, institutions, and constituencies that must be navigated in the process of negotiation and collaboration. In the case of a large federal system like Ethiopia, with many donors—each with their own set of priorities and constituencies to answer to—it is necessary to understand the nuanced structural and political conditions which influence reform design in order to examine the conditions under which Ethiopia has been able to develop a suite of education reforms aimed at improving quality with equity, as well as for identifying potential barriers to successful implementation.

**Figure 1:**
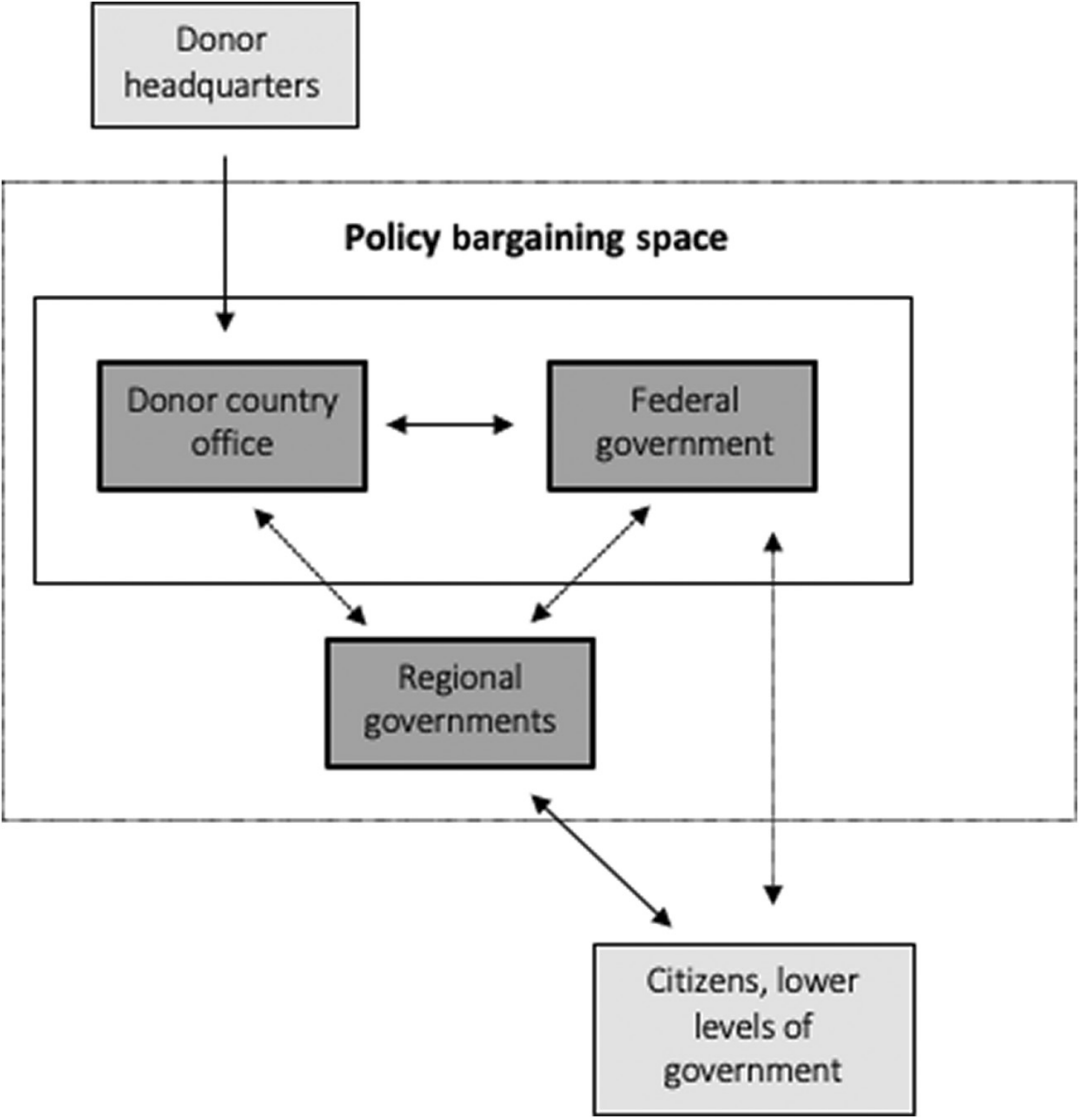
Aid policy bargaining in Ethiopia.

We draw on Swedlund’s (2017) framework for aid‐policy bargaining and compromise to explore donor–government and within‐government dynamics in the design process of GEQIP‐E, paying particular attention to how actors influence the policy design process. Figure 1 illustrates our adapted version of Swedlund’s framework for the Ethiopian education context, showing the stylized two‐way flows of influence over policy decisions. We acknowledge that donors are accountable to their broader organizational goals and incentives, but they must also adapt priorities based on country‐specific goals and dynamics. This leads to a process of negotiation and bargaining between country‐based donors and government actors. As such, the two boxes inside the solid line are where the primary policy negotiations take place.

Swedlund and Lierl (2019) suggest that for both strategic and normative reasons, donors often push for an inclusive aid‐bargaining process, that may involve local governments, civil society, or other relevant actors. However, this inclusivity can reduce the power of central governments in the negotiation process. As a result, Swedlund & Lierl (2019) find that governments are sometimes willing to trade influence over policy priorities in exchange for reducing the demands of inclusivity during the negotiation process. We find this dynamic to be relevant in Ethiopia as it raises questions about the inclusion of regional governments and other local education actors in the design phase of GEQIP‐E.

## 3 GLOBAL AND NATIONAL INFLUENCES ON EDUCATION PLANNING IN ETHIOPIA

Since 2015, the SDGs have reflected an explicit focus on equitable and inclusive quality and equity in education. Ethiopia’s national development policies have historically mirrored global agendas, although often with a stronger focus on both equity and quality than earlier global frameworks, as seen in the 1994 Education and Training Policy, and each of the associated Education Sector Development Plans (ESDPs) that have been in place since 2000 (Yorke, Rose, & Pankhurst, 2021).

Aligned with Ethiopia’s ESDPs, the GEQIP reforms were first introduced in 2008, with objectives to co‐ordinate donor support to the education sector; to improve teaching and learning conditions in primary and secondary school; and to strengthen education institutions and service delivery at federal and regional levels (World Bank, 2008). In its third iteration, the broad principles set out in 2008 generally remain relevant. GEQIP‐E, starting in 2018, has however placed explicit emphasis on improving learning outcomes, with particular attention to girls, children with disabilities, and pastoralist communities. The intention is that the GEQIP design occurs through a collaborative process primarily between the federal Ministry of Education, regional education bureaus (REBs), and donors. GEQIP focuses on those areas within the overarching national education strategies set out in the ESDPs, which are viewed as integral for achieving quality in primary schooling and improving equity. GEQIP‐E aligns with Ethiopia’s fifth ESDP (2015–2020). It shows the considerable convergence between priority areas with respect to primary schooling, with the exception of information and communication technology (ICT), which is not included in GEQIP‐E. In addition, cross‐cutting issues beyond the school system, such as school health and nutrition, are reflected in ESDP V, but have not been included in GEQIP‐E. We explore potential reasons for these divergences arising from the government–donor negotiation processes in our analysis.

Ethiopia has been one of the world’s largest recipients of international development aid since the fall of the Derg regime in 1991 (Feyissa, 2011; Lie & Mesfin, 2018). Between 2007 and 2017, Ethiopia received nearly USD 3.6 billion in international aid for education, making it the largest recipient of aid for education on the African continent.^2^ In education, the largest donors to Ethiopia in 2017 were the United Kingdom (36%), World Bank (33%), United States (12%), Germany (6%), France (2%), and Japan (2%). The main donors to GEQIP pooled funds include the World Bank, UNICEF, the governments of the United Kingdom, Finland, and Norway, and the Global Partnership for Education, with a total contribution of about USD 110 million over ten years. Of this total, the funds to GEQIP comprise the majority of total aid allocated to basic education. As such, questions arise about the extent of donor influence and country ownership on the focus of GEQIP reforms.

The Ethiopian government has been described as a “strong” aid‐recipient government, exercising greater negotiating capital with donors than many other countries in sub‐Saharan Africa. Earlier work suggests that this in part due to a sense of “Ethiopian exceptionalism,” demonstrated by Ethiopia’s record on economic development and poverty reduction, potentially giving the country greater bargaining power (Clapham, 2018; Furtado & Smith, 2008). Ethiopia’s political environment, characterized by a strong developmental state, also shapes the government’s approach to aid bargaining (Clapham, 2018). Moreover, its current role as the location of the African Union (AU) and the United Nations Economic Commission for Africa (UNECA) give it a sense of status within the region (Clapham, 2018; Lie & Mesfin, 2018).

Education has often been a space of convergence for donors and the government in Ethiopia, in that priorities across actors tend to align (Furtado & Smith, 2008). There are, however, examples of areas of contention. One notable divergence has been the relative balance between spending on primary and tertiary education: donors have commonly been of the view that domestic spending on tertiary education is too high given the status of primary and secondary completion, while government has continuously defended this spending (Gruber & Kosack, 2014).

## 4 DATA COLLECTION AND ANALYSIS

We conducted 81 semi‐structured, key informant interviews during two stages of fieldwork with all stakeholders who were involved in the design of GEQIP‐E. Stage 1 included interviews with senior officials from all donor organizations which contribute to GEQIP as a pooled fund and director‐level federal government officials from the Ministries of Education and Finance (February 2018). During stage 2, interviews were conducted with senior regional‐level stakeholders (Stage 2, May 2018) (see Table 1). As a federal system, regions in Ethiopia are semi‐autonomous and are responsible for the implementation and oversight of education policies. The geographic, socio‐economic, and ethnic diversity of the nine regions can result in different education constraints and thus varying priorities. Regional officials therefore added critical insight into the extent of their ownership and influence over the design of GEQIP‐E. Our sample included regional officials from seven of Ethiopia’s nine regions, excluding Gambella and Afar due to security concerns at the time of interviews.

**Table 1:** Key stakeholder interviews, Stages 1 and 2

Fieldwork stage	Stakeholder groups	Number of interviews
Stage 1 February 2018	Donors	8
	Federal‐level stakeholders	12
	City administration stakeholders: Addis Ababa	7
	Regional‐level stakeholders: Oromia	5
Stage 2 May 2018	Regional‐level stakeholders: Amhara	10
	Regional‐level stakeholders: Benishangul Gumuz	10
	Regional‐level stakeholders: SNNPR	11
	Regional‐level stakeholders: Somali	8
	Regional‐level stakeholders: Tigray	10
		81

We adopted a multi‐stage, purposive sampling strategy to select participants, beginning with an actor‐mapping exercise to identify key stakeholders involved in the design and implementation phases of GEQIP II, and/or the design of GEQIP‐E. The purposive strategy facilitates a “*critical case*” analysis in which the selected participants are critical to understanding the phenomenon of interest, in this case the design of the GEQIP programme (Onwuegbuzie & Collins, 2007, pp. 37–38). The actor‐mapping work was conducted during an initial round of interviews with donors and Ministry of Education (MoE) officials in December 2017. This enabled us to identify key informants across stakeholder groups, namely: a) multilateral and bilateral donors that provide financial and/or technical assistance to GEQIP; b) federal stakeholders from key directorates in the MoE Education; and c) regional stakeholders from REBs. Our data collection coincided with the end of the second phase of GEQIP (GEQIP II), and with preparations for the implementation the third phase of the reforms (GEQIP‐E). This provided an opportunity to learn about the key individuals, institutions, and power dynamics which shaped the design and priorities of GEQIP‐E.

Semi‐structured interviews were conducted by members of the RISE Ethiopia team.^3^ Interview protocols were customized for each stakeholder group to ensure that topics covered were relevant and based on each participant’s area of expertise. Interviews broadly covered stakeholders’ roles and responsibilities, and their perspectives on issues such as: a) the design of GEQIP‐E, including individual and organizational involvement; b) formal and informal processes for including/excluding stakeholders’ priorities; c) GEQIP II implementation, including overall challenges and successes; and d) lessons from GEQIP I and II for GEQIP‐E. In an attempt to ensure the anonymity of interview participants, we have not identified specific donors, as this is likely to make it possible to identify individuals.^4^ We have also been mindful of these potential sensitivities in the way that we present the analysis.

Interviews were digitally recorded and transcribed. Where interviews were conducted in a language other than English (e.g. Amharic or Afan Oromo), they were translated during transcription. We used both inductive and deductive approaches to qualitative analysis, in which patterns, themes, and categories were identified using our conceptual framework (for example, relating to ownership of the GEQIP reforms), but attention was also paid to unanticipated themes which emerged from the data (for example, limited capacity within the system; Srivastava & Hopwood, 2009). Coding was facilitated using NVivo software.^5^

## 5 FINDINGS

Our analysis of the GEQIP‐E negotiation process suggests that, overall, the design of the reforms was collaborative between the federal government and donors, in which neither party monopolized ownership over the process, but that ownership was leveraged differently across actors. We find that the federal government maintains control over policy priorities and final design owing to its political capital. At the same time, donors influence negotiations through their financial and social capital and are thus able to shape specific features of reform. We find that regional officials, who are at the forefront of implementing reforms, have had limited influence or ownership over the GEQIP‐E design process.

### 5.1 Shared ownership facilitated by political capital is associated with a common commitment to global and national agendas, and builds on previous engagements

The SDGs have provided a common starting place for identifying shared priorities for donors and governments. When asked about the key factors influencing the shift in focus on quality linked to learning outcomes with equity in GEQIP‐E, donor and government representatives indicated that these elements of GEQIP‐E at least partly reflect the shared interest of the Ethiopian government and donors in aligning national programmes with international priorities, in particular related to the SDGs:

…ultimately GEQIP is aligned with the SDGs [which are] very universal, talking about equitable quality education […] so there is no [difference], I mean, [between] emerging and developed regions […] And it is also not going to be like [an] MDG donor‐recipient country [relationship]. […] So there is no way that Ethiopia would say no, no, no this is not my priority, this is the [donor], because this is what Ethiopia as a nation signed as member states signed, when SDG was endorsed (MoE representative).

One way in which government officials exercised ownership over negotiation processes was by setting reform boundaries through linking GEQIP‐E with existing overarching national plans and strategies. Specifically, GEQIP‐E priorities were drawn from the government’s ESDP which ensured that any goal reflected in GEQIP‐E would necessarily reflect a government education priority. Donor, government, and regional education bureau representatives emphasized that the federal government maintained primary ownership and strong negotiating power over the priorities reflected in the final reform. We found that in some cases, ministry officials reported a stronger sense of ownership than donors perceived them to have had in the process because government officials felt they had set the parameters within which further negotiations took place (see also Asgedom, et al., 2019). One donor official stressed the importance of the government’s involvement, while a regional representative expressed confidence that the design process was led by the government:

The role of the Ministry is that they are the most important partner, and if they were not the most important partner [to other donor organisations], we wouldn’t support it; because this is our way of working directly with the government, instead of having a bilateral agreement (Donor representative).

I know GEQIP was entirely prepared by our government. It was prepared by the MoE [under] the then‐Minister of Education […] The nation requested to [an international donor] that it has this package and needs support. Then [donor] brought [other] donors (REB representative, Benishangul Gumuz).

While GEQIP was not “*entirely prepared by*” the government as the REB representative suggests, this quote indicates a belief that the government played a strong role in setting the reform agenda and the parameters for further reform negotiations.

The process of negotiation for the new reform was also foregrounded by the building of trust and a common agenda in previous phases of GEQIP reforms. Notably, stakeholder interviews suggest that the conditions for effective donor–government co‐ordination during GEQIP‐E were established in earlier phases of the GEQIP reforms. These conditions facilitated further collaboration during the GEQIP‐E design phase, in support of a new, more complex goal of achieving equitable learning for all. At the same time, shortcomings of earlier iterations of the GEQIP programme helped shape the terms and priorities of reform efforts across actors.

As noted, across the different phases of GEQIP, the stated aim has been to strengthen the quality of education service delivery in Ethiopia (World Bank, 2008, 2013), but the specific approach to improving quality has evolved over time. The main aim of GEQIP I was to create the conditions for learning within the Ethiopian education system, primarily through an inputs‐based approach. GEQIP II built on GEQIP I and introduced a focus on “serving the most under‐served regions” (World Bank, 2013, p. 8), but stopped short of setting learning targets, which include monitoring of progress towards equity.

Overall, there is consensus among stakeholders that the “inputs” focus of GEQIPs I and II filled a critical need in the education system, and that the reforms—specifically through the reliable flow of financial resources directly to schools through the school grant component—had the potential to strengthen the quality of education by providing resources that schools had the discretion to allocate according to their needs. Some regional stakeholders suggested student learning outcomes had improved thanks to changes related to teacher quality and availability of textbooks. By contrast, other regional stakeholders refer to evidence that questions the relationship between improvements in the quality of inputs and student learning outcomes:

As you know the government has taken a number of interventions using GEQIP II money in terms of revising the curriculum materials, provision of trainings for teachers, production of textbooks and teacher guide. However, the changes reported in the learning outcomes of students are not still promising ‐ this has been confirmed by the EGRA [Early Grade Reading Assessment] results. This shows that there is a missing link [between interventions and learning outcomes] (REB representative, Oromia).

These concerns are consistent with evidence of low levels of learning in Ethiopia (Iyer et al., 2020). It suggests that, while earlier GEQIP reform efforts have built a viable mechanism for policy collaboration between donors and the MoE, the results have fallen short.

Ownership has also evolved and become strengthened following successful collaboration. In comparison to previous iterations of GEQIP, one donor representative suggested that the GEQIP‐E design process involved the government to a greater degree than previous GEQIP processes:

I was not [involved] in the design of GEQIP II […] but [during] the process of implementation I felt a little bad. There was a lot of bulldozing by [certain donors]. So, I imagined that the development [of GEQIP II] was in a similar way. But it is different with GEQIP [E] […] there was […] joint consultations among the rest of the [donor] partners pushing back in terms of questions, pushing in terms of actually looking at [programme] documents and revising the documents and contributing to the document[s]. […] So [I] must say that it is much better than GEQIP II (Donor representative).

### 5.2 Negotiations over specific reforms are shaped by the relative influence of financial and social capital across donors

While we find evidence of collaboration and shared goals between government and donors, this did not mean that actors always agreed on specific reform priorities. Donors may be constrained by their respective organizational mandates and also vary in their individual priorities. To navigate these constraints, different donors leveraged different resources—notably relative financial and social capital—to push for their individual priorities to be reflected in reform design.

GEQIP’s pooled‐fund approach meant that negotiations took place between the government and a collective body of donors, rather than bilaterally through individual donor–government arrangements. This has benefits as it has the potential to strengthen efficiency and align donor funding behind common priorities. As mentioned, however, it can also complicate negotiation processes where individual donors need to answer to their respective headquarters and, in the case of bilateral donors, their taxpayer bases. This can at times result in conflicting priorities among donors. This dynamic can lead to a multi‐layered negotiation process in which donors must negotiate both with the government and with each other to find consensus on policy priorities. Illustrating this, an MoE representative reflected on the implications of competing priorities among donors for the Ethiopian government:

Every [donor] country has its own assistance strategy, and every penny is a taxpayer’s payment. And if it is not behind, you know, the country assistance strategy, it is not good way of spending money. For Ethiopia you cannot say, “I don’t want [the] World Bank, I want DFID”. You have to address all issues, I mean, very diplomatically, because we are […] [a] very poor country. We need [every] single pennies [sic] to be used for the intended purpose. So, we accept all, as government. That has to be handled as, you know […] the Ministry of Education is co‐chairing the [Education Technical] Working Group, and has to balance the intervention[s] coming from the World Bank, DFID and other donors (MoE representative).

This MoE representative acknowledges the pressures on donors to justify how they spend taxpayers’ funds on international aid, but also characterizes Ethiopia’s position as a disadvantageous one due to its status as a “*very poor country*” which needs every single penny on offer from donors. This can put the government in a position of having to balance often competing donor demands. Donors recognize this dynamic as well. As one donor representative discusses, different donors push for priorities that reflect their own interests:

[Donor One] for instance wants to focus on special needs education. [Donor Two] [wants to focus on] gender, I mean, there are also interests in other result areas. Donor Three, in addition to everything they want to focus on […] the teacher profession in our programme design […] Donors have their own interests, but you cannot really include everything they ask, we have to really come together at some point ( Donor representative).

As such, the government and donor perspectives suggest that, while the government maintains high‐level ownership of the overall design process, negotiations over specific details often take place between individual donors in which the government adopts a more mediating than direct bargaining role.

Two areas in which donor and government partners struggled to reach consensus were the inclusion of ICT‐related activities and school‐feeding programmes. One donor representative suggested that a larger donor was reluctant to invest further in ICT for schools, because there was no evidence that their investments in ICT via earlier GEQIP iterations (around USD 25 million to date) had led to a positive effect on learning outcomes—thus limiting confidence that ICT would prove to be a worthwhile investment in GEQIP‐E. The same donor representative asserted that the exclusion of ICT from GEQIP‐E could be explained by dominant donors’ opposition to further investment in the area, combined with the fact that other donors “weren’t as passionate [about ICT] as they were with textbooks or school leadership.” A government official noted, however, that in the absence of donor funding, the government could still choose to fund ICT on its own, thus raising questions about whether government is willing to accept compromise where there are other avenues to ensure its priorities are met.

The inclusion of school feeding was also identified as being more contentious, particularly due to its close links to improving equity for the most marginalized. In contrast to evidence of the weak links between ICT and equity or learning outcomes, the evidence on links between school feeding and equity are well documented (for example, Evans & Mendez Acosta, 2021). Interviews with the federal government officials as well as regional education officials in Addis Ababa and Oromia suggest that stakeholders across levels of government pushed for school feeding directed at poor families to be included in GEQIP‐E, citing evidence linking the positive effects of school feeding with encouraging enrolment of more disadvantaged students, and preventing dropout. For example:

Students from poor families cannot attend classes properly with an empty stomach. Therefore, economic opportunities for the families must be expanded. In GEQIP‐E, school feeding programs must be taken as a primary area of intervention (REB representative, Addis Ababa).

An official from the MoE further suggests that school feeding is one way to particularly attract students with intellectual disabilities, who are among the most disadvantaged, to school:

Intellectual disabilities are the more disadvantaged because even I have told you that when they allot money they allocate small money, but they need a lot of money because even [school] feeding is one of the methods of attraction of these children. Through [school] feeding you see first we attract them [to school] and second, we teach them daily living, doing language skills, cleaning, washing, and so on you see. But support for these children is very small.

Donors ultimately decided that school feeding was too costly to be funded through GEQIP‐E. As a representative of a smaller donor suggests, this decision to exclude it was driven primarily by larger donors, which leveraged their financial capital to prevent it from being funded through GEQIP‐E:

The main focus is school feeding because the children need to be helped. If not, they don’t learn. It is as simple as that. And addressing the emerging regions when they have specific needs is now part of GEQIP‐E… [But] school feeding is absolutely not being funded by the [major donors]. I don’t know why, you have to ask [them] (Donor representative).

In the face of multiple competing priorities, in both these cases, larger donors were able to use their financial capital to push for influence over specific strategies and priorities to exclude. This influence was recognized by donors themselves:

I think there was also ganging up on certain things and pushing the agenda forwards. Some of the partners felt that the [larger donors] were not listening there were focus that would came together and agree on positions and push that position until the [larger donors] listen and include the ideas that were coming up and even take direction that was being suggested (Donor representative).

Thus, while the government had a final say over the programme as a whole thanks to its political capital, our findings suggest that some larger donors used their relative financial power to influence the inclusion and exclusion of specific reform components:

The issue is that, this is a donor pool fund [and] the lion’s share comes from [certain donors] and [on] the capacity of negotiating of the Ministry and the region, particularly the MoE, I do have some reservation[s]. Sometimes donors will come with their own agenda and try to enforce (Donor representative).

I think the MoE itself [was most influential]. Because eventually, they have to agree on everything. They have to approve it because we cannot shove anything down their throat at all… So, the biggest influence is supposed to be them. But we also know that the ones who dig deeper into their pockets sometimes have a real final say (Donor representative).

Donors that contributed smaller amounts were also able to influence the reform process, by leveraging social capital through their established relationships with the government, drawing also on their technical expertise to shape reform design. These are perhaps the kinds of roles we might expect donors to assume during reform negotiation processes. However, the disproportionate influence of external technical expertise can also increase donor influence over specific activities. One donor describes their role as a technical expert and with a focus on ensuring that the government is accountable to global commitments:

So, when a programme as big as GEQIP is designed, [the] technical expertise […] we provide is our own input in terms of the designing of the program itself to ensure that what is designed is acceptable to the MoE framework but is also in line with what we are taking in terms of the SDGs and so forth. […] We are in the meetings to ensure that what is said is applicable to the partners of the MoE but also at the international level, you know, that you are driving the same agenda (Donor representative).

We found evidence that some smaller donors had become influential because they had established a track record of leadership in the education sector, co‐ordinating activities; particular areas of expertise; and, in some cases, direct contact or relationships with regional stakeholders. In addition, some of these smaller donors also had long‐term, established engagement on specific issues, such as disability and early childhood education. These on‐going, close partnerships added to donors’ ability to act as a credible and trusted partner of the government, which they could leverage to strengthen their voice in negotiations with other, often larger, donors. This in turn strengthened their negotiating capital in the policy design process, despite comparatively smaller financial contributions to the overall fund. One such donor discussed their role co‐chairing a taskforce which focused on services for people with disabilities, spanning government, donor, and non‐governmental organization (NGO) partners. This sustained and cross‐sectoral involvement in one thematic area helped lend the donor greater credibility to push for disability as a priority in the education sector as well. In another example, one donor representative described how they were able to maintain influence during negotiations despite their comparatively small financial contributions to GEQIP‐E:

It is crudely saying it is, but it’s buying a place on the table […] So, everybody recognises our technical importance. But we also need to put on the litmus. Our money is very little. We contribute the least. But because we sit on the table and we have the technical capacity, we bring in much more than the money can buy. That brought us to the table. Right now, on GEQIP‐E we feel we are contributing a lot on the quality component; on the equity component in terms of the programme that we have managed to influence. That decision was a conscious decision. We had a challenge at the beginning (Donor representative).

Thus, for some donors, the ability to influence GEQIP‐E was driven more by social capital associated with long‐standing, credible commitment; a continued country presence; technical engagement; and a relationship with the government, rather than the level of financial resources they provided.

### 5.3 Bureaucratic hierarchies in a developmental state can limit inclusivity of within country ownership

While federal government and donor priorities largely converged through their negotiations, engagement by regional officials was limited. One education official from an emerging region expressed frustration over the perception of the level of influence donors exercised over the reform:

Yes, I […] participated in the design of GEQIP‐E. We met together and aligned GEQIP along with ESDP V. To my dismay […] I noticed the concept note that compares and analyses the implementation of GEQIP II with the upcoming GEQIP‐E was prepared by [a donor]. [The donor] took the leading [sic] in the design of GEQIP‐E. I strongly believe that this [should] be entirely done by MoE, for it is the major sector and owner of GEQIP (REB representative, Benishangul Gumuz).

Bureaucratic hierarchies in a developmental state come with trade‐offs in the reform negotiation process. This political environment simplified negotiations by limiting the bargaining space to donors and the federal government. In doing so, it resulted in the exclusion of actors responsible for the implementation, and ultimately the success, of the reform. In the context of GEQIP‐E, we find that the central government negotiates with donors largely independently from other domestic actors, including regional government officials (see also Asgedom et al., 2019). While this is likely to have simplified the design process, it also contributed to a sense of exclusion among regional officials. Regional officials who were more aware of GEQIP‐E—typically specific individuals working in regional GEQIP co‐ordination offices—were notably critical of the design process, and in particular, the failure to adapt the programme’s design according to regional priorities:

I realised that in GEQIP‐E, donor interest prevailed. I do not think GEQIP‐E was designed taking into consideration the real problems on the ground (REB representative, Addis Ababa).

While participatory consultation workshops were held for regional and local governments to contribute to the design of GEQIP‐E, many felt that these activities were ceremonial in nature, while real, substantive engagement in the policy process took place primarily between donors and the central MoE. In addition, officials from emerging regions expressed frustration over disproportionate exclusion compared to other wealthier regions.

No one has participated in the design process from this region. After [GEQIP‐E] is designed we were invited to a workshop to familiarise us with GEQIP‐E […] but the design was already prepared by consultants and we were invited to the workshops. We gave comments on issues which are not considered in the design and which are not emphasised (Regional Government stakeholder).

Furthermore, local‐level stakeholders—including *woreda* (district) education bureau officials, schools, teachers, parents and students—were identified by both donor and government stakeholders as having had the lowest level of influence in the design of GEQIP‐E. In particular, teachers and parents were identified as having had extremely limited involvement (Asgedom et al., 2019). To some extent, this may be expected in a federal system with centralized decision‐making, though it can result in sub‐optimal decision‐making to meet the needs of diverse regions. This characteristic of the policy environment and negotiation process could have important implications for the success of GEQIP reforms, as regional and local‐level governments are ultimately responsible for policy adoption and implementation.

This is reflected in the dissatisfaction with the government’s role in the GEQIP‐E negotiation process that some regional government officials expressed. Several REB officials felt that their top priorities were not sufficiently reflected and suggested that this was because the process was donor‐led. However, given the broad exclusion of regional government officials, few were fully informed of who was actually involved in the decisions. It is possible that central government officials allowed regional actors to channel their frustrations toward donors to deflect blame for a non‐inclusive reform process. Several stakeholders described a consultation meeting held by a donor in late 2017 to present plans for GEQIP‐E to regional representatives, at which several regional officials reportedly challenged donors on their priority areas. According to donor representatives present at this meeting, one point of disagreement related to the exclusion of an ICT component from GEQIP‐E:

[One REB official] was making a very, very passionate argument about the technology: “Ethiopia cannot afford to miss out [on] technology. The fact that we are poor does not mean we cannot access technology. It is not a privilege [for] the rich”, you know. […] [REB officials were] making a demand on donors […] in terms of what their expectation was, what they want to see in this programme (Donor representative).

While this may look like donor domination from the perspective of a regional official, it may in fact be a result of the federal government choosing to negotiate with donors on its own, rather than necessarily being a result of donors’ domination of the process. Alternatively, one may also take this at face value, as donors’ failure to recognize and respond to the priorities of recipient stakeholders.

## 6 DISCUSSION

In countries in which donors provide financial and technical support, questions of ownership become important to understanding the policy bargaining process. Based on our findings in the context of Ethiopia’s quality education reform, we argue that ownership over the policy‐making process should be understood as a dynamic process in which each party leverages different forms of capital—whether political, financial, or social—to influence reform design. This aligns with the arguments by Whitfield and Fraser (2008b) and Swedlund and Lierl (2019) of negotiation as a process. It also highlights that the concept of “ownership,” which features prominently in the Paris Declaration and Accra Agenda, may manifest in different forms, and to varying degrees, within countries as reform environments and as partnerships evolve.

More specifically, in Ethiopia, we find that the federal government leverages political capital by setting the parameters of possible priorities through its education‐sector plans, determining the boundaries of the negotiation process, and brokering negotiations across donors. Donors in turn influence specific strategies within the reform package through their financial and social capital. Larger donors leverage their financial capital to exclude selected activities that are potentially costly, notably ICT and school feeding. Smaller donors draw on their social capital through the strength of existing relationships with government coupled with technical expertise to ensure the inclusion of specific areas in the programme, namely early childhood education and disability (arguably areas that government also supported). These areas of disagreement or misalignment appear to be driven in part by differences in the use of evidence and by external constraints set by donors’ organizational mandates rather than necessarily from donors simply throwing their financial weight around to monopolize ownership.

At the same time, we find that the history of the GEQIP programme, aligned with global agendas associated with the SDGs and the government’s existing education plans, has provided an important common starting point. Notably, previous experience in joint reform efforts through earlier iterations of GEQIP provided the building blocks and a critical sense of mutual credibility and trust across actors, thus paving the way for more ambitious and complex reform efforts. Put simply, focusing on inputs in earlier phases of GEQIP with successful collaboration between government and donors laid the technical, political, and relational building blocks for the pursuit of more ambitious education reforms, focused on quality with equity. While this seems to have been effective overall in the Ethiopia case, government–donor negotiations may function in a more one‐sided manner in places without a history of past collaboration of this kind.

As in many contexts, larger donors hold the “power of the purse” to some degree. However, our evidence suggests that they did not fully control the process. As such, the design phase of GEQIP‐E was characterized by a largely collaborative process of negotiation and compromise between and among donors and the federal government. Notably, the terms of the negotiation and collaboration were set by the federal government, drawing on its political capital. As a strong developmental state, the federal government also manages a hierarchical bureaucracy in which they can exclude or limit other actors from the policy bargaining process, in this case the regional governments within Ethiopia.

While limiting negotiations to the federal government and donors perhaps makes agreement on complex quality and equity‐focused reforms potentially more straightforward, we also caution that this could exclude critical voices from the design process and consequently limit the success of the reforms in the long run. While our findings cannot confirm whether the exclusion of regional government officials was intentional or strategic on the part of the federal government, we find little evidence of efforts to substantively include them in the process despite clear demand from REBs to be included. This is despite there being legitimate reasons to include REBs as they operate as semi‐autonomous governments and are ultimately responsible for the implementation and success of reforms. The recent escalation of regional conflict within the country could further illustrate the importance of national and regional collaboration in a federal system.

The pooled nature of GEQIP funding has resulted in parallel negotiation processes both among actors within the donor group, as well as between donors as a collective group and the government, in which all actors try to use their respective bargaining power to shape reforms. This pooled funding approach, which is a common way of organizing aid finance in social sectors in many countries, may have contributed to the reduced influence of additional actors at the regional level. The need to navigate competing demands, combined with the challenges of pursuing a complex reform striving towards achieving quality with equitable across diverse regional contexts—each of which faces different challenges to varying degrees—raises questions regarding the likely success of GEQIP‐E in the absence of additional measures to build buy‐in and capacity at lower levels. At the same time, we are reminded that making major changes, like those involved in the process of pursuing reforms for quality with equity, is a difficult undertaking that will require time, adjustment, and continuous improvement along the way.

As Swedlund (2017) has noted, the current aid literature overwhelmingly focuses on allocation and effectiveness. However, to date, academic literature has rarely examined how donors and recipient countries engage in compromise, and the factors that drive perceptions of credibility and processes for negotiation. Swedlund argues that it is critical to understand these dynamics before we can evaluate the effectiveness or sustainability of a given approach or reform. The findings discussed in this article contribute to addressing this gap by exploring the complex processes of multi‐stakeholder policy and reform negotiation in a strong developmental state. This may function differently in more participatory states in which civil society and local governments may be able to punish the government for their exclusion through political consequences, such as incumbent voting. Understanding and engaging with the complexity of these processes is crucial if large‐scale reforms in Ethiopia and other resource‐constrained education systems in low‐ and lower‐middle‐income countries are to ultimately achieve equitable access and learning for all. Finally, our analysis also raises interesting normative questions for future research, relating to whose voice *should* count: within the government, who is included, who is excluded, and who decides?

## FUNDING INFORMATION

The RISE programme is supported by funding from the United Kingdom’s Foreign, Commonwealth and Development Office (FCDO), the Australian Government’s Department of Foreign Affairs and Trade (DFAT), and the Bill and Melinda Gates Foundation.

## Data Availability

The data are not publicly available due to privacy or ethical restrictions.

## Footnotes

^1^
Including the Paris Declaration (2005), the Accra Agenda for Action (2008), the Busan Partnership Agreement (2011), and the Global Partnership for Effective Development Co‐operation’s (GPEDC) Nairobi Outcome Document (2016).

^2^
Calculated using the OECD Development Assistance Committee Credit Reporting System (DAC CRS, 2019). The amount reflects gross disbursements in constant 2016 dollars.

^3^
The RISE Ethiopia team involved in this study comprises researchers from the Institute of Educational Research, Addis Ababa University, and the REAL Centre, University of Cambridge, UK.

^4^
In some circumstances, it may be preferable not to anonymise elite interviews of the kind we conducted. This is in part because it can be important to know the specific position of each of the individuals for the purposes of the analysis. In addition, it can sometimes be difficult to truly anonymise data given that it may be possible to work out the identity based on information provided, and thus it could be risky to promise anonymity. However, we are aware that some issues are raised that may be considered sensitive, and so have sought to keep identities anonymous.

^5^
Interview protocols and the code list are available on request.

## References

[dpr12634-bib-0001] Asgedom, A. , Hagos, B. , Lemma, G. , Rose, P. , Teferra, T. , Wole, D., & Yorke, L. (2019). Whose influence and whose priorities? Insights from government and donor stakeholders on the design of the Ethiopian General Education Quality Improvement for Equity (GEQIP‐E) Programme. Research on Improving Systems of Education (RISE) Programme insight note. https://riseprogramme.org/publications/whose‐influence‐and‐whose‐priorities‐insights‐government‐and‐donor‐stakeholders‐design

[dpr12634-bib-0002] Booth, D. (2012). Aid effectiveness: Bringing country ownership (and politics) back in. Conflict, Security & Development, 12(5), 537–558 10.1080/14678802.2012.744184

[dpr12634-bib-0003] Bruns, B. , Macdonald, I. H. , & Schneider, B. R. (2019). The politics of quality reforms and the challenges for SDGs in education. World Development, 118, 27–38. 10.1016/j.worlddev.2019.02.008

[dpr12634-bib-0004] Clapham, C. (2018). The Ethiopian developmental state. Third World Quarterly, 39(6), 1151–1165 10.1080/01436597.2017.1328982

[dpr12634-bib-0005] Evans, D. K. , & Mendez Acosta, A. (2021). Education in Africa: What are we learning? Journal of African Economies, 30(1), 13–54 10.1093/jae/ejaa009

[dpr12634-bib-0006] Feyissa, D. (2011). Aid negotiation: The uneasy “partnership” between EPRDF and the donors. Journal of Eastern African Studies, 5(4), 788–817 10.1080/17531055.2011.642541

[dpr12634-bib-0007] Furtado, X. , & Smith, W. J. (2008). Ethiopia: Retaining aid sovereignty in aid relations. In L. Whitfield (Ed.), The politics of aid: African strategies for dealing with donors (pp. 131–155). Oxford University Press.

[dpr12634-bib-0008] Gruber, L. , & Kosack, S. (2014). The tertiary tilt: Education and inequality in the developing world. World Development, 54, 253–272 10.1016/j.worlddev.2013.08.002

[dpr12634-bib-0009] Hickey, S. , & Hossain, N. (2019). The politics of education in developing countries: From schooling to learning. Oxford University Press.

[dpr12634-bib-0010] Iyer, P. , Rolleston, C. , Rose, P. , & Woldehanna, T. (2020). A rising tide of access: What consequences for equitable learning in Ethiopia? Oxford Review of Education, 46(5), 601–618 10.1080/03054985.2020.1741343

[dpr12634-bib-0011] Kosack, S. (2012). The education of nations: How the political organization of the poor, not democracy, led governments to invest in mass education. Oxford University Press.

[dpr12634-bib-0012] Lie, J. H. S. , & Mesfin, B. (2018). Ethiopia: A political economy analysis. Norwegian Institute of International Affairs.

[dpr12634-bib-0013] Menashy, F. (2019). International aid to education: Power dynamics in an era of partnership. Teachers College Press.

[dpr12634-bib-0015] Ministry of Education . (2015). *Education Sector Development Plan V (ESDP V), 2015/6 – 2020/1. Program Action Plan*. https://www.globalpartnership.org/sites/default/files/2016‐06‐ethiopia‐education‐sector‐plan‐vi_0.pdf

[dpr12634-bib-0017] National Planning Commission , & United Nations Ethiopia . (2015). Assessment of Ethiopia’s progress towards the MDGs . Millennium Development Goals Report, (pp. 1–103). https://reliefweb.int/report/ethiopia/ethiopia‐millennium‐development‐goals‐report‐2014‐assessment‐ethiopia‐s‐progress

[dpr12634-bib-0018] National Planning Commission . (2016). *Growth and Transformation Plan II (GTP II) 2015/16 – 2019/20*. Volume I: Main Text. Government of Ethiopia. https://www.cabri‐sbo.org/en/documents/growth‐and‐transformation‐plan‐ii‐gtp‐ii‐2015‐16‐2019‐20‐volume‐i‐main‐text

[dpr12634-bib-0019] Onwuegbuzie, A. J. , & Collins, K. M. (2007). A typology of mixed methods sampling designs in social science research. Qualitative Report, 12(2), 281–316. 10.46743/2160-3715/2007.1638

[dpr12634-bib-0021] Seid, Y. , Taffesse, A. S. , & Ali, S. N. (2016). Ethiopia: An agrarian economy in transition. In H. Bhorat & F. Tarp (Eds.), Africa's lions: Growth traps and opportunities for six African economies (pp.37–76). Brookings Institution Press.

[dpr12634-bib-0022] Srivastava, P. , & Hopwood, N. (2009). A practical iterative framework for qualitative data analysis. International Journal of Qualitative Methods, 8(1), 76–84 10.1177/160940690900800107

[dpr12634-bib-0023] Swedlund, H. J. (2017). The development dance: How donors and recipients negotiate the delivery of foreign aid. Cornell University Press.

[dpr12634-bib-0024] Swedlund, H. J. , & Lierl, M. (2019). The rise and fall of budget support: Ownership, bargaining and donor commitment problems in foreign aid. Development Policy Review, 38(1), 50–69 10.1111/dpr.12463

[dpr12634-bib-0025] UN Department for Economic and Social Affairs . (2018). Sustainable Development Goal, 4. https://sdgs.un.org/goals/goal4

[dpr12634-bib-0027] Whitfield, L. , & Fraser, A. (2008a). Introduction: Aid and sovereignty. In L. Whitfield (Ed.), The politics of aid: African strategies for dealing with donors (pp. 1–26). Oxford University Press.

[dpr12634-bib-0028] Whitfield, L. , & Fraser, A. (2008b). Negotiating aid. In L. Whitfield (Ed.), The politics of aid: African strategies for dealing with donors (pp. 27–44). Oxford University Press.

[dpr12634-bib-0029] World Bank . (2008). Program appraisal document: General Education Quality Improvement Program Phase I. World Bank.

[dpr12634-bib-0030] World Bank . (2013). Program appraisal document: General Education Quality Improvement Program Phase II. World Bank.

[dpr12634-bib-0031] World Bank . (2017). Program appraisal document: General Education Quality Improvement Program for Equity (GEQIP‐E). World Bank.

[dpr12634-bib-0032] World Inequality Database on Education . (2018). https://www.education‐inequalities.org.

[dpr12634-bib-0033] Yorke, L. , Rose, P. , & Pankhurst, A. (2021). The influence of politics on girls’ education in Ethiopia. In P. Rose , M. Arnot , R. Jeffery , & N. Singal (Eds.), Reforming education and challenging inequalities in southern contexts: Research and policy in international development. Routledge.

